# Comparative Analysis of Evolutionarily Conserved Motifs of Epidermal Growth Factor Receptor 2 (HER2) Predicts Novel Potential Therapeutic Epitopes

**DOI:** 10.1371/journal.pone.0106448

**Published:** 2014-09-05

**Authors:** Xiaohong Deng, Xuxu Zheng, Huanming Yang, José Manuel Afonso Moreira, Nils Brünner, Henrik Christensen

**Affiliations:** 1 Chongqing Key Lab of Catalysis & Functional Organic Molecules, Chongqing Technology and Business University, Chongqing, China; 2 Section of Molecular Disease Biology, and Sino-Danish Breast Cancer Research Centre, Institute of Veterinary Disease Biology, Faculty of Health and Medical Sciences, University of Copenhagen, Copenhagen, Denmark; 3 Institute of Veterinary Disease Biology, Faculty of Health and Medical Sciences, University of Copenhagen, Copenhagen, Denmark; 4 Beijing Genomics Institute and Sino-Danish Breast Cancer Research Centre, Shenzhen, China; H. Lee Moffitt Cancer Center & Research Institute, United States of America

## Abstract

Overexpression of human epidermal growth factor receptor 2 (HER2) is associated with tumor aggressiveness and poor prognosis in breast cancer. With the availability of therapeutic antibodies against HER2, great strides have been made in the clinical management of HER2 overexpressing breast cancer. However, de novo and acquired resistance to these antibodies presents a serious limitation to successful HER2 targeting treatment. The identification of novel epitopes of HER2 that can be used for functional/region-specific blockade could represent a central step in the development of new clinically relevant anti-HER2 antibodies. In the present study, we present a novel computational approach as an auxiliary tool for identification of novel HER2 epitopes. We hypothesized that the structurally and linearly evolutionarily conserved motifs of the extracellular domain of HER2 (ECD HER2) contain potential druggable epitopes/targets. We employed the PROSITE Scan to detect structurally conserved motifs and PRINTS to search for linearly conserved motifs of ECD HER2. We found that the epitopes recognized by trastuzumab and pertuzumab are located in the predicted conserved motifs of ECD HER2, supporting our initial hypothesis. Considering that structurally and linearly conserved motifs can provide functional specific configurations, we propose that by comparing the two types of conserved motifs, additional druggable epitopes/targets in the ECD HER2 protein can be identified, which can be further modified for potential therapeutic application. Thus, this novel computational process for predicting or searching for potential epitopes or key target sites may contribute to epitope-based vaccine and function-selected drug design, especially when x-ray crystal structure protein data is not available.

## Introduction

Human epidermal growth factor receptor 2 (HER2) is one of four members of the EGF receptor family of receptor tyrosine kinases that mediate cell proliferation, differentiation and survival [1]. Overexpression of HER2, resulting from amplification of the *ErbB2* gene, is observed in approximately 20% of breast cancers, and amplification of HER2 significantly correlates with increased disease aggressiveness and thereby with poor patient outcome [2], [3], [4], [5], [6]. Overexpression of HER2 can be detected in the early stages of breast cancer, and it is maintained in the progression to metastatic disease [7], [8], indicating that HER2 has an important effect on breast cancer progression. As a result, HER2 has become a critical therapeutic target in the treatment of breast cancer patients.

Trastuzumab, a monoclonal antibody directed against the extracellular domain of HER2, which consists of four domains (domain I, II, III and IV) [9], is currently the first choice of treatment for HER2-positive breast cancer patients, as it improves overall survival and reduces the risk of disease recurrence when administered in combination with chemotherapy (for review see [10]). Nevertheless, not all HER2 positive patients benefit from Trastuzumab treatment [11] and around 15% of breast cancer patients relapse after an initial response to trastuzumab-based therapy, suggesting that *de novo* or acquired resistance to trastuzumab has developed [12]. Thus, additional therapeutic agents are necessary in the treatment of HER2-positive breast cancer patients, with the aim of improving survival.

Pertuzumab is another humanized monoclonal antibody that binds to the extracellular domain II of HER2, the dimerization arm [13], thereby blocking signaling transduction that results from dimerization with other members of the EGFR family [14]. Although pertuzumab had low clinical efficacy when used alone, it has an excellent effect in HER2-positive breast cancer patients when used in combination with trastuzumab [15], [16], [17]. Pertuzumab administrated in combination with trastuzumab and docetaxel significantly prolongs the progression-free survival without increased cardiac toxic side effects in metastatic breast cancer patients [18], [19]. These data suggest that an additive or perhaps synergistic effect can be achieved using several antibodies directed against different epitopes of the same protein (HER2) [19].

Another therapeutic strategy to block HER2 makes use of small molecule tyrosine kinase inhibitors (TKIs), such as the dual EGFR/HER2 TKI lapatinib [20], [21]. Unfortunately, as it is the case for other molecular targeted therapies, the clinical responses to lapatinib tend to be short-lived. However, several lines of evidence suggest continued dependence of HER2+ breast cancers on HER2 signaling network after progression on anti-HER2 therapy (reviewed in [22]), providing a rationale for multilayered HER2 blockade. Therefore, searching for additional epitopes/targets of HER2 is needed to broaden clinical selection and improve the efficacy of anti-HER2 treatment. Currently, the use of three-dimensional (3D) structural data combined with some experimental approaches such as pepscan, phage display, or mutagenesis scanning, are the gold standard of epitope-based vaccine design [23], [24]. But 3D data are not available for all proteins, and experimental approaches are expensive and time-demanding techniques. Thus, computational processes that could function as a compensational approach to predictably identify some desirable epitopes or functional targets for rational vaccine or drug design are badly needed.

In the present study, we report a novel computational process used to predict functional motifs of HER2, which matched very well with epitopes of trastuzumab and pertuzumab (HER2's antibodies).

## Materials and Methods

### Data collection and protein BLAST (basic local alignment search tool)

The human HER2 amino acid sequence with accession number P04626 was compared to the UNIPROT database using BLAST via the web service (http://www.uniprot.org/) with default setting.

### Structural conserved motifs scanning in PROSITE database

According to the domain organization of the ErbB family, the extracellular domain region of HER2 has ∼620 residues [25]. The human extracellular domain of HER2 (1-620) amino sequence was uploaded in the PROSITE Scan online service (http://prosite.expasy.org/scanprosite/). The databases used for structurally conserved motifs scanning included UniProtKB/Swiss-Prot, splice variants and UniProtKB/TrEMBL databases. The searching results with low level scores were also allowed to show in the output. The other parameters used the default setting.

### Searching for fingerprints in the PRINTS database

The amino sequence of the extracellular domain of HER2 (ECD HER2) was aligned for fingerprints in web service (http://www.bioinf.man.ac.uk/cgi-bin/dbbrowser/fingerPRINTScan/FPScan_fam.cgi) with default settings.

### Comparison of structurally conserved motifs with the epitopes of HER2's antibodies based on the crystal 3D structure

The crystal structure of the extracellular domain of the human HER2 complexed with trastuzumab Fab (PDB ID: 1N8Z) and the structure of HER2-pertuzumab complex (PDB ID: 1S78) were obtained from NCBI (http://www.ncbi.nlm.nih.gov/structure/?term=HER2). Using Cn3D software, the structurally conserved fragments of ECD HER2 were superposed and labeled on the two crystal structures. Furthermore, as supplementary reference to our prediction, FoldX (http://foldx.crg.es/) were introduced with in silico mutagenesis 'repair module', which allowed to predict the amino acids involved in the binding of HER2 to the antibodies by alanine mutation based on 'force field' statistical considerations [26].

## Results

### The HER2 amino acid sequence was highly conserved in mammals

We hypothesized that functional structure motifs could be conserved and maintained during evolution. In order to determine the level of homology in the HER2 amino acid sequence among mammals, available orthologs of HER2 were searched by BLAST in the UniProt database. The result showed that the HER2 protein sequence indeed was highly homologous across species with over 80% similarity in mammals, including horse (*Equus caballus*), pig (*Sus scrofa*), mouse (*Mus musculus*), rat (*Rattus norvegicus*), and cow (*Bos taurus*) (Table 1). This indicated that some meaningful sequences/structures of ECD HER2 protein existed among these conserved amino acid sequences.

**Table 1:** The identity of human HER2 protein in the UniPort database.

Enter accession number	Organism	Protein names	Length	Identity
P04626	Homo sapiens (Human)	Receptor tyrosine-protein kinase erbB-2	1255	100%
G3QPD2	Gorilla gorilla gorilla (Lowland gorilla)	Uncharacterized protein	1258	99%
K7DJM0	Pan troglodytes (Chimpanzee)	V-erb-b2 erythroblastic leukemia viral oncogene homolog 2, neuro/glioblastoma derived oncogene homolog (Avian)	1255	99%
H2QCV1	Pan troglodytes (Chimpanzee)	Uncharacterized protein	1247	99%
G1QK40	Nomascus leucogenys (Hylobates leucogenys)	Uncharacterized protein	1255	99%
P04626-4	Homo sapiens (Human)	Isoform 4 of Receptor tyrosine-protein kinase erbB-2	1240	99%
I0FM22	Macaca mulatta (Rhesus macaque)	Receptor tyrosine-protein kinase erbB-2 isoform a	1255	98%
G7NI22	Macaca mulatta (Rhesus macaque)	Receptor tyrosine-protein kinase erbB-2	1255	98%
P04626-5	Homo sapiens (Human)	Isoform 5 of Receptor tyrosine-protein kinase erbB-2	1225	100%
F6WAF2	Callithrix jacchus (White-tufted-ear marmoset)	Receptor tyrosine-protein kinase erbB-2 isoform a (Uncharacterized protein)	1255	96%
G7PUM4	Macaca fascicularis (Cynomolgus monkey)	Receptor tyrosine-protein kinase erbB-2	1231	98%
H2NU95	Pongo abelii (Pongo pygmaeus abelii)	Uncharacterized protein	1256	97%
F7CBG7	Callithrix jacchus (White-tufted-ear marmoset)	Uncharacterized protein	1239	96%
F6UJQ9	Callithrix jacchus (White-tufted-ear marmoset)	Uncharacterized protein	1225	97%
F6VNY4	Equus caballus (Horse)	Uncharacterized protein	1258	94%
Q49LT4	Felis catus (Cat) (Felis silvestris catus)	Epidermal growth factor receptor type 2	1260	94%
F1PIQ9	Canis familiaris (Dog) (Canis lupus familiaris	Receptor tyrosine-protein kinase erbB-2	1260	93%
H0X3G4	Otolemur garnettii (Garnett's greater bushbaby)	Uncharacterized protein	1256	93%
H9BB15	Felis catus (Cat) (Felis silvestris catus)	Erb2	1260	93%
G1SZL0	Oryctolagus cuniculus (Rabbit)	Uncharacterized protein	1255	93%
M3WCC2	Felis catus (Cat) (Felis silvestris catus)	Uncharacterized protein	1260	93%
H9BNW8	Ursus americanus (Euarctos americanus)	V-erb-b2 erythroblastic leukemia viral oncogene-like protein 2	1260	92%
G1LTV6	Ailuropoda melanoleuca (Giant panda)	Uncharacterized protein	1263	92%
O18735	Canis familiaris (Dog) (Canis lupus familiaris)	Receptor tyrosine-protein kinase erbB-2 (EC 2.7.10.1) (Proto-oncogene c-ErbB-2) (p185erbB2) (CD antigen CD340)	1259	92%
I3M2X8	Spermophilus tridecemlineatus (Ictidomys tridecemlineatus)	Uncharacterized protein	1259	93%
M3YU62	Mustela putorius furo (Mustela furo)	Uncharacterized protein	1260	92%
F1MCQ7	Bos taurus (Bovine)	Uncharacterized protein	1257	92%
W8FW17	Tupaia chinensis (Chinese tree shrew)	ERBB2	1255	92%
A8WED5	Canis familiaris (Dog) (Canis lupus familiaris)	HER-2	1242	93%
M3X794	Felis catus (Cat) (Felis silvestris catus)	Uncharacterized protein	1260	92%
D2I2F8	Ailuropoda melanoleuca (Giant panda)	Putative uncharacterized protein	1236	92%
G3SLF2	Loxodonta africana (African elephant)	Uncharacterized protein	1257	90%
K7GS43	Sus scrofa (Pig)	Uncharacterized protein	1231	91%
S7MLI8	Myotis brandtii (Brandt's bat)	Receptor tyrosine-protein kinase erbB-2	1227	91%
G3H5Y0	Cricetulus griseus (Cricetulus barabensis griseus)	Receptor tyrosine-protein kinase erbB-2	1256	89%
S9YT31	Camelus ferus (Wild Bactrian camel)	Receptor tyrosine-protein kinase erbB-2	1253	90%
F1RWM5	Sus scrofa (Pig)	Uncharacterized protein	1234	88%
Q8K3F9	Rattus norvegicus (Rat)	Neu protooncoprotein	1259	88%
F1LRR9	Rattus norvegicus (Rat)	Receptor tyrosine-protein kinase erbB-2	1257	88%
P06494	Rattus norvegicus (Rat)	Receptor tyrosine-protein kinase erbB-2	1257	88%
W5PQJ8	Ovis aries (Sheep)	Uncharacterized protein	1251	89%
Q60553	Mesocricetus auratus (Golden hamster)	Receptor tyrosine-protein kinase erbB-2	1254	88%
P70424	Mus musculus (Mouse)	Receptor tyrosine-protein kinase erbB-2	1256	88%
H0V6A0	Cavia porcellus (Guinea pig)	Uncharacterized protein	1236	88%
J3QLU9	Homo sapiens (Human)	Receptor tyrosine-protein kinase erbB-2	1055	99%
G1PRB8	Sarcophilus harrisii (Sarcophilus laniarius)	Uncharacterized protein	1244	85%
G3WQQ2	Sarcophilus harrisii (Sarcophilus laniarius)	Uncharacterized protein	1255	81%
F7C962	Monodelphis domestica (Gray short-tailed opossum)	Uncharacterized protein	1257	80%

The table only shows resemblance to HER2 higher than 80%.

### The ECD HER2 protein sequence contained three evolutionally conserved structural motifs

To determine the structurally conserved motifs in the ECD HER2 protein, the protein sequence from M1 to E620 was uploaded in Prosite ExPASy. We obtained three hits (Table 2).

**Table 2:** The structurally conserved motifs of ECD HER2 detected by PROSITE Scan.

Predicted Conserved motifs	Amino Acid Residues	Predicted domain (condition)	Contact region
F1 (L244-K311; PDB sequence number.)	L**H**CPALVT**Y**NTDT**F**ESMPNPEGRYTFGASCVTACPYNYLSTD**V**G**S**CTLVCP**LH**NQEVTAEDGTQRCEK	Sushi (Disulfide 246C-x-293C and 277C-x-309C)	Heterodimerization region and Pertuzumab binding region.
F2 (N549-E558; PDB sequence number.)	NGSVTCFG**PE**	NHL	Trastuzumab interacting region.
F3 (D570-E598; PDB sequence number.)	**DPPF**CVARCPSGVKPDLSYMPIW**KFPDEE**	ZF-THAP type, degenerate	Trastuzumab interacting region.

The amino acids in bold present involved residues in epitopes of antibodies: trastuzumab and pertuzumab.

The first hit, Fragment 1 (F1), was predicted as a type of Sushi domain by the presence of four conserved cysteine residues (C246-C293 and C277-C309), forming two disulfide bounds [27], which covered amino acid residues from L244 to K311 according to the PDB sequence number.

The second hit, Fragment 2 (F2), was predicted as a NHL domain, defined by amino acid homological presenting in three proteins: NCL-1, HT2A, and LIN-41[28], which was located from N549 to E558 (PDB sequence number). Finally, the third hit, Fragment 3 (F3), was predicted as a degenerated zinc finger THAP-type domain (C2H2) [29], [30], which embraced 29 amino residues from D570 to E598 (PDB sequence number) (Table 2). The zinc finger HTAP degenerate domain mapped to the N-terminal location of a sequence-specific DNA-binding factor [31], [32].

### Three structurally conserved fragments are located in functional domains of ECD HER2

**Figure 1:**
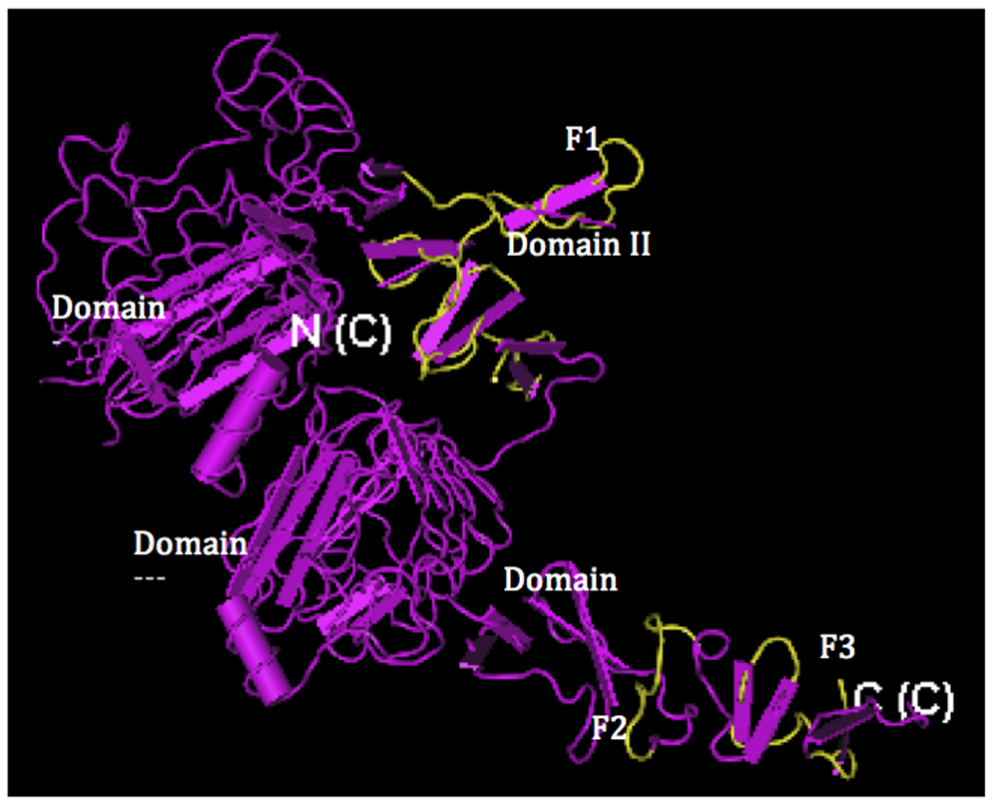
The location of the three structurally conserved motifs (F1, F2 and F3) on ECD HER2 crystal structure. Purple: ECD HER2 receptor; Yellow: structurally conserved motifs (F1, F2 and F3). N(C) and C(C) are ECD HER2's N-terminal and C-terminal, respectively.

In order to explore the superposition of those structurally conserved fragments with functional domains of ECD HER2, the predicted fragments were superposed on the crystal structure of ECD HER2 (Figure 1) [33]. F1 (L244-K311) was completely located at center of domain II (G200-R329), which formed the exposed dimerization arm of HER2, suggesting that the predicted fragment F1 involved in HER2 dimerization and hereby could affect the activation of HER2. F2 (N549-E558) and F3 (D570-E598) were located at domain IV (W499-N607) which was the arm extending to domain II and further mediating the activation of HER2. Trastuzumab worked well in clinic via its interaction with this domain [33]. The results indicated that these two predicted fragments are matched with HER2's functional domains and probably they were critical sites for HER2's activation. (To keep consistency with the crystal structure study [33], we used the PDB residues number here, rather than the sequential residues number of HER2.)

### The structurally conserved fragments matched well with epitopes of HER2 antibodies

**Figure 2:**
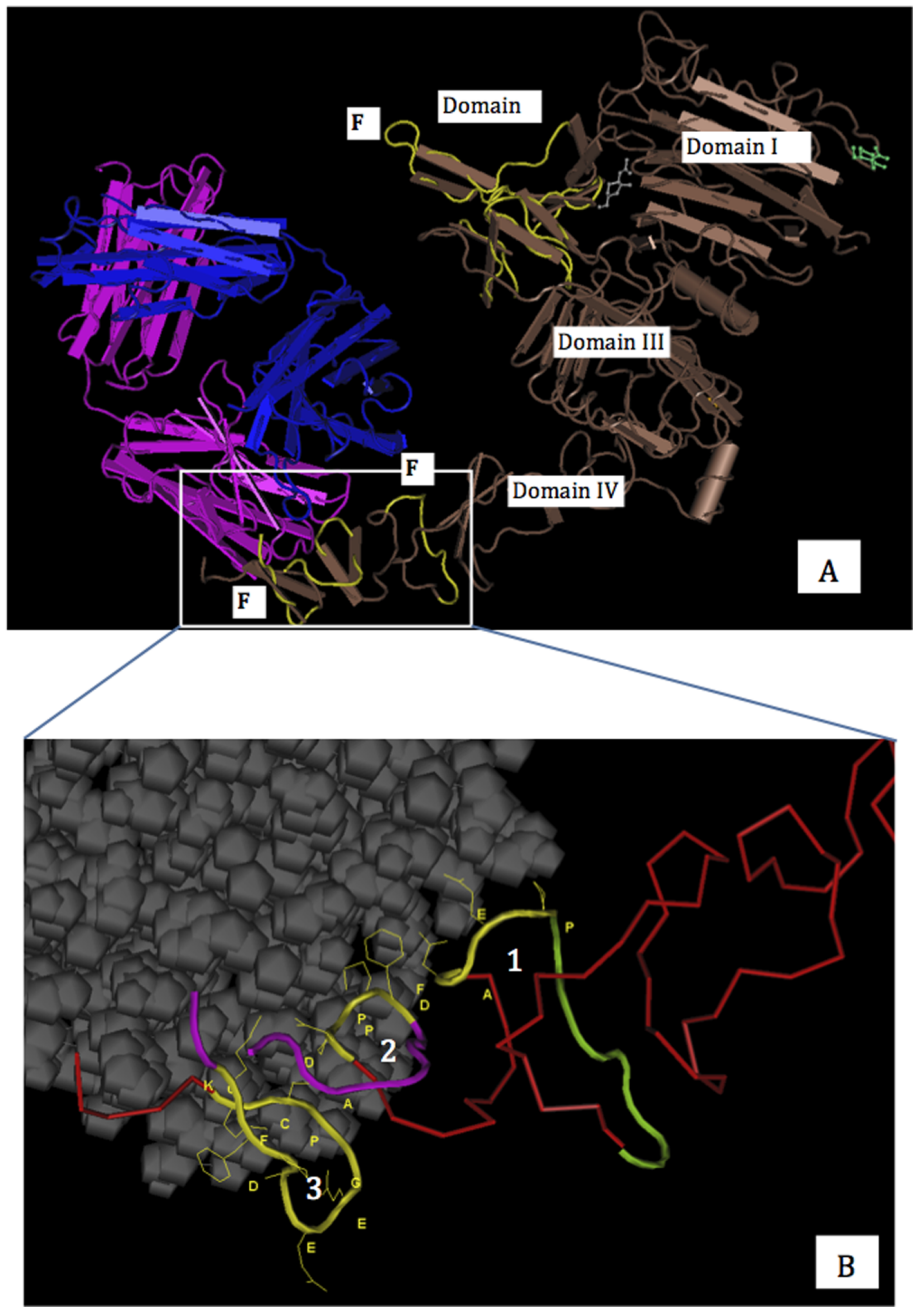
Comparison of structurally conserved motifs of ECD HER2 with the epitope of trastuzumab. A: The location of F1, F2 and F3 in the tube worm representation of the HER2-trastuzumab complex. HER2 is colored dark brown and trastuzumab is in chain form: light chain in bright purple and heavy chain in blue. The structurally conserved motifs (F1, F2 and F3) are highlighted in yellow. B: Enlarged view of binding interface between ECD HER2 and trastuzumab. Trastuzumab is shown in grey, with a space-fill protein backbone and side chains style. The numbers indicted in the figure show the three loop regions (highlighted in yellow) mediating the interaction with trastuzumab. F2 is shown in light green and F3 is in bright purple. The overlapped residues between F2, F3 and interaction loops are P557 and E558 (F2, on loop 1 (P557-D561)), D570, P571, P572 and F573 (F3, on loop 2 (D570-F573)), K593, F594, P595, D596, E597 and E598 (F3, on loop 3 (K593-P603)). According to the crystal structure of HER2-trastuzumab complex [33], there are eight residues invisible in the F3 region. The numbers shown in the figure are consistent with PDB data rather than sequential numbering for HER2 residues.

To investigate whether the predicted structurally conserved motifs/fragments could be key regions for the biological function(s) of HER2, they were compared with epitopes of HER2 antibodies based on the HER2: trastuzumab [33]/HER2: pertuzumab [14] co-crystal structures, respectively. The result showed that the conserved F1 did not locate in the interaction surface with trastuzumab, whereas the F2 and F3 were involved in the interface of trastuzumab binding (Figure 2A). According to Cho et al., three loops in the domain IV of HER2 are involved in the HER2-Trastuzumab binding surface [33] (Figure 2). We found the two residues of F2 P557 and E558 were located in loop 1 (P557-D561). The four residues of F3 D570, P571, P572 and F573 were positioned in loop 2 (D570-F573), and the residues of F3 K593, F594, P595, D596, E597 and E598 were found in loop 3 (K593-P603) (Figure 2B). The results showed the predicted F2 and F3 partly consisted of the epitope of trastuzumab, implying the predicted F2 and F3 probably are the pivotal regions for HER2's activation.

**Figure 3:**
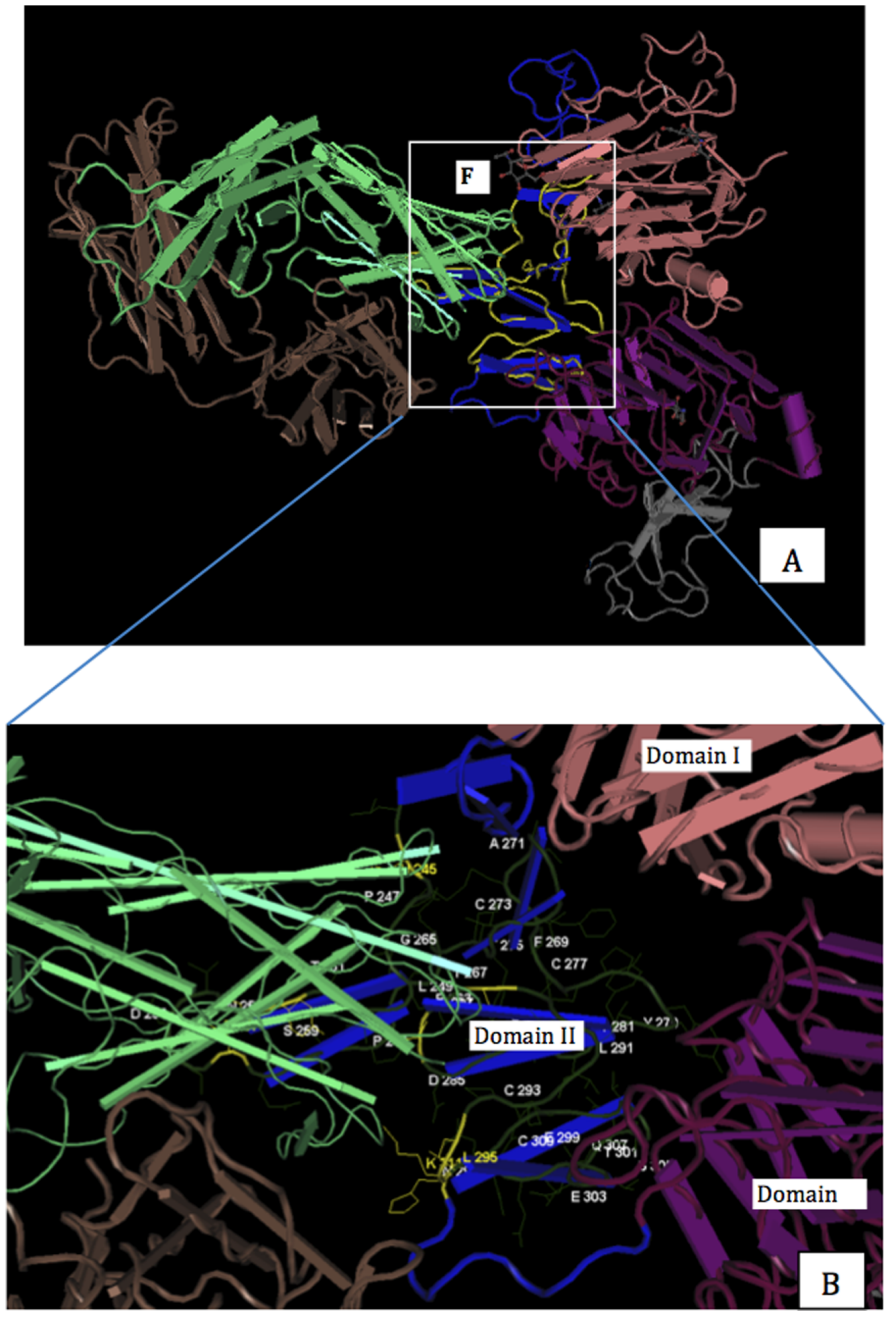
Comparison of structurally conserved motifs of ECD HER2 with the epitope of pertuzumab Fab. A: The location of F1 in the tube worm representation of the HER2-pertuzumab complex. According to Franklin, M.C. et al. [14], ECD HER2 is colored according to domain: domain I in pink, domain II in blue, domain III in dark purple and domain IV in grey. Pertuzumab is shown as a chain: light chain in green and heavy chain in dark grey. The highlighted in yellow shows the location of structurally conserved F1. B: The enlarged view shows the binding interface between ECD HER2 and pertuzumab. Domain II is shown in blue, and the F1 is shown as a tube worm with wire side-chains in dark green. The residues involving in the interaction with pertuzumab [14] are highlighted in yellow. The numbers shown in the figure are consistent with PDB data rather than sequential numbering for HER2 residues.

The structural conserved F1 was completely embedded in the center of domain II (in blue, Figure 3A), which has been revealed as the interface with pertuzumab [14]. Furthermore, we found that the directly interacted residues of HER2 with pertuzumab, H245, Y252, F257, K311 and H296, and important residues of HER2, H245, V286, S288, L295, H296 and K311, for affinity with pertuzumab [14] were located in the predicted conserved F1 (Figure 3B, showed in dark green). This result suggested that the predicted conserved F1 contained pertuzumab's epitopes.

All these structural comparisons results between the predicted conserved fragments and epitopes of HER2's antibodies implied that the structurally/functionally conserved fragments of HER2 probably contain more potentially powerful epitopes/target sites.

### The ECD HER2 protein sequence contained some conserved fingerprints

For purpose of combination of linear feature and structural characterization of epitopes to reduce the prediction error, the PRINTS database, another deterministic pattern, was introduced. And then, 9 top fingerprints were significantly screened from the ECD HER2 sequence with p-value<0.01 (Table 3). Among them, the fingerprints ANATRNSFRASE 280, BACEL 290, and TRANSYNTHGLU 316 overlapped with F1; the fingerprints 4FE4SFRDOXIN 577 partly overlapped with F2; the fingerprints ALPHATUBULIN 600 and 4FE4SFRDOXIN 595 overlapped with F3 (The position number is consistent with ECD HER2 amino acid number).

**Table 3:** The linearly conserved motifs (fingerprints) of ECD HER2 searched by PRINTS.

Fingerprint Name	P-value	Position	Sequence	Matched fingerprints with structurally conserved motifs
ALPHATUBULIN	9.2×10^−6^	439	GAYSLTLQGLGIS	
		600	*CPSGVKPDLSYMPI* *	F3
ANATRNSFRASE	4.1×10^−5^	79	EVQGYVLIAHNQVRQVPLQRLRIV	
		280	*ESMPNPEGRYTFGASCVTACPYNY* *	F1
ONCOGENEAML1		110	DNYALAVLDNGDPLNNTTPVTG*	
	0.00021	374	LAFLPESFDGDPASNTAPLQ	
FMOXYGENASE		125	NTTPVTGASPGGLRELQ	
	0.00048	354	RAVTSANIQEFAGCKKIF	
BASICPTASE		236	CHEQCAAGCTG	
	0.00052	529	VNCSQFLRGQECVEEC	
4FE4SFRDOXIN		577	FGPEADQCVACA*	F2
	0.0013	595	*FCVARCPSGVKP* *	F3
BACE1		113	ALAVLDNGDPLNNTTPVTGAS	
	0.0023	290	*TFGASCVTACPYNYLSTDVG*	F1
TRNASYNTHGLU	0.0033	177	NQLALTLIDTNRSR*	
		316	*PLHNQEVTAED* *	F1
ANIONEXCHNGR	0.0020	11	LLLALL	
		251	DCLACLHF*	

The fingerprints overlapped with structurally conserved motifs, but not present in the interface of HER2: antibodies were showed in italics. Underlined residues present key sites involved in epitopes of trastuzumab and pertuzumab.

*indicates that this motif affected the interaction energy between HER2 and pertuzumab and Fab trastuzumab in 1S78 and 1N8Z 3D structures, respectively.

### Some of both linear and structural conserved regions were beyond the epitopes of HER2's antibodies

To further explore the match between the linear and structural conserved regions and epitopes of HER2's antibodies (trastuzumab and pertuzumab), they were compared. Some linear and structural conserved overlapped regions, including fingerprints ALPHATUBULIN 600, ANTRNSFRASE 280, 4FE4SRDOXIN 595, BACE1 290 and TRNASYNTHGLU 316, did not present in the interface of HER2:antibodies (Table 3. shaded fingerprints). The result showed that the predicted structurally conserved fragments contained some linearly conserved segments, suggesting that those segments could be other efficient epitopes or drug target sits. In order to evaluate the effect of those conserved motifs listed in Table 3 on binding energies to pertuzumab and Fab trasutumab, FoldX was introduced as a supplementary reference. The result showed that some amino acids in the overlapped regions of structurally and linearly conserved motifs were predictively involved in the interaction with trastuzumab and pertuzumab (Table 3), implying our prediction performance was reliable.

## Discussion

Great achievements have been obtained with antibody-based therapies in the treatment of cancer [34]. Trastuzumab and pertuzumab, two antibodies targeting the HER2 protein, are good examples of antibody-based therapies. Clinical studies have shown that these two monoclonal antibodies, which are directed against different epitopes of HER2, display an additive/synergistic anti-tumor effect when they are used in combination with docetaxel in the treatment of metastatic breast cancer, even though some patients developed resistance [15], [16], [19]. Hence, it is conceivable that using several antibodies toward the same molecular target results in an additive/synergistic effect, since the antibodies are immunologically generated towards different parts of the antigen. Therefore, more efficient antibodies against HER2 could provide more options for treatment and greater benefits for HER2-positive breast cancer patient than those presently in use.

The identification of the most efficient epitopes/target sites, as the first step for epitope-based vaccine/drug design, is still an unsolved problem. In the present study, we hypothesized that evolutionary structurally conserved fragments of the ECD of HER2 contain efficient druggable epitopes/targets.

We used the PROSITE database that can detect biologically meaningful inter-domains, based on manually derived alignments and extensive manually curated documentation [35], to predict structurally conserved motifs. We also introduced the PRINTs database which is a compendium of protein motifs with a series of conserved regions of aligned sequences, since the linear segment is another essential part of protein epitopes [36]. We found that all predicted fragments were located in the important dimerization arms regions of HER2: within domain II and IV. Comparing the structurally conserved fragments of HER2 with epitopes of trastuzumab and pertuzumab, respectively, we further found that the predicted fragments were involved in the formation of epitopes for trastuzumab and pertuzumab, which are the most efficient known antibodies against HER2. Thus, our results showed that the evolutionary structurally conserved fragments of HER2 contain novel epitopes/targets, not being targeted by trastuzumab or pertuzumab, which strongly supports our hypothesis.

Furthermore, considering that the linear conserved motifs most probably have a meaningful role in the formation of druggable epitopes, we combined the two kinds of motifs and compared the results generated. Some of these segments were not presented in the epitopes of trastuzumab and pertuzumab, but included in the structurally conserved fragments. The result implies that these segments could be potential new epitopes/targets of ECD HER2 for peptide-based vaccine and drug design. Hence, this novel computational process provides a new complementary approach for efficiently selecting critical regions/sites as epitope for drug targeting.

Numerous studies have shown that HER2 is involved in many fundamental cellular processes, including cell migration, cell survival and cell proliferation and differentiation (for review [1]), suggesting that the basal function of HER2 is probably shared in mammals. Indeed, our results confirmed that the homology of HER2 amino acid sequence is very high in mammal species with over 80% similarity. Thus, it is reasonable to suggest that some similar functional inter-structures exist in the HER2 protein. Three-conserved inter-structure motifs of ECD HER2 (named as F1, F2 and F3) were identified as best hits using the PROSITE Scan. The first two, F1 and F2, corresponding to the Sushi and NHL domains in the ECD of HER2, respectively, are linked to protein-protein interaction, protein-binding modules and cell adhesion, all of which are coherent with the function of HER2, a growth factor receptor protein [37], [38], [39], [40], [41].

However, the third domain, F3, was predicted as a degenerated zinc finger HTAP domain, which has DNA-binding capability. It is rather intriguing that the trans-membrane part of HER2 should contain a DNA-binding domain, even if HER2 reportedly can function as a transcriptional regulator [42]. Studies have demonstrated that HER2 is involved in COX2

[43], and ribosomal RNA gene [44] transcriptional regulation and HER2 can be located to the nucleus, where transcriptional activity takes place reasonably [42]. In addition, a truncated HER2 has also been reported to be located to nucleus and to contribute to acquired resistance to HER2 kinase inhibitors [45], [46]. Taken as a whole these lines of evidence suggest that a HER2 may function as a transcriptional regulator, in which case a DNA-binding domain is not a remarkable finding.

We found that all identified fragments, based on their predicted structural features, can be rationalized to match the activation of HER2. Hence, this prediction approach probably is a believable method for drug design, especially when one selects a protein or activity of a protein to inhibit.

To add bio-function assessment to the predictive model, in the present study, the correspondence between localization of predicted fragments and HER2's functional domains was studied using co-crystal structure of ECD HER2: antibody.

The X-ray crystal structure of ECD HER2 has revealed that the domain II of HER2, plays a key role in the activation of the receptor, through which contacting and forming dimerization with other ErbB family members [33], [47]. According to our hypothesis, some of evolutionally conserved fragments should be included in domain II of HER2. Indeed, F1, as predicted as Sushi domain, located in the center of domain II and completely overlapped with interaction surface with pertuzumab [14]. Furthermore, Franklin et al. revealed by a crystallographic study that the residues H245, Y252, F257, H296 and K311 interact with pertuzumab directly, and the residues H245, H296, K311, V286, S288 and L295 significantly mediated the affinity of HER2 to pertuzumab. Without exception, we also found that all these pivotal residues located in the predicted F1, as expected. This further confirms that the structurally conserved motif F1 probably has more efficient, potential epitopes/targets.

Results from X-ray structure analysis have shown that domain IV of HER2 is another important domain for activation of HER2, being involved in receptor-receptor contact [48], [49], suggesting that the domain IV should contain a receptor-receptor contacting structure. Our prediction showed that a NHL structure in F2, which has been proposed to be a protein-protein interaction structure [39], [41], exists in domain IV. Furthermore, the X-ray crystal structure of the ECD HER2 complex with trastuzumab has also revealed that domain IV of HER2 is the domain of epitope of trastuzumab, by its' loop 1 (P557-D561), loop 2 (D570-F573), and loop 3 (K593-P603) to contact trastuzumab [33]. Similarity, our predicted fragments F2 and F3 partly formed the epitope of trastuzumab, which meet our expectations.

Since the linear peptides are the basis of forming epitopes, we introduced the PRINTScan tool to scan the linear conserved segment and compared these with structurally conserved motifs. We found that some fingerprints also appeared in the rest residues of the structurally conserved motifs (highlighted in Table 2). These results indicate that the overlapped regions in structurally and linearly conserved motifs could be potentially efficient epitopes/targets for a new vaccine or drug design.

By and large, one can distinguish three main mechanism of trastuzumab resistance: (1) functional bypass of blockade by either up-regulation of downstream signaling or equivalent alternate pathways, (2) failure to elicit immune-mediated killing of tumor cells, and (3) hindrance of antibody binding to HER2. With respect to the latter, our study may provide cues for novel molecular mechanisms of resistance by pinpointing important biological epitopes of ECD HER2. Conversely, the approach presented here may provide the means to identify potentially druggable epitopes/targets that can bypass known impediments to antibody binding to HER2, such as expression of a constitutively active, truncated form of HER2 that lacks the ECD, and consequently the binding site of trastuzumab, or masking of trastuzumab cognate epitopes by steric hindrance of HER2 by cell surface proteins [45], [50], [51].

In conclusion, by comparing the structurally conserved motifs of ECD HER2 protein with epitopes of trastuzumab and pertuzumab, we confirmed our hypothesis that evolutionally structural conserved motifs of the ECD HER2 protein contain potentially druggable epitopes/targets. Furthermore, on the basis of a comparison between the structurally conserved motifs and the linearly conserved motifs, we proposed that some segments being potentially more important biological epitopes of ECD HER2 can be further modified for potential therapeutic application. In the present study, we also provide a novel procedure to predict or search for potential epitopes or key target sites in proteins, which may contribute to the design of epitope-based vaccines and drugs. In particular, when there is no X-ray crystal structure available, it is easy to narrow the searching region by means of this process, sharply reducing the interesting residues.
